# Longitudinal Assessment of Working Memory Performance in the APPswe/PSEN1dE9 Mouse Model of Alzheimer’s Disease Using an Automated Figure-8-Maze

**DOI:** 10.3389/fnbeh.2021.655449

**Published:** 2021-05-13

**Authors:** Fran C. van Heusden, Sara Palacín i Bonsón, Oliver Stiedl, August B. Smit, Ronald E. van Kesteren

**Affiliations:** Department of Molecular and Cellular Neurobiology, Center for Neurogenomics and Cognitive Research, Amsterdam Neuroscience, Vrije Universiteit Amsterdam, Amsterdam, Netherlands

**Keywords:** figure-8-maze, longitudinal behavioral assessment, APP/PS1 mice, Alzheimer’s disease, working memory

## Abstract

Alzheimer’s disease (AD) is a progressive neurodegenerative disorder, with a long preclinical and prodromal phase. To enable the study of disease mechanisms, AD has been modeled in many transgenic animal lines and cognitive functioning has been tested using several widely used behavioral tasks. These tasks, however, are not always suited for repeated longitudinal testing and are often associated with acute stress such as animal transfer, handling, novelty, or stress related to the task itself. This makes it challenging to relate cognitive dysfunction in animal models to cognitive decline observed in AD patients. Here, we designed an automated figure-8-maze (F8M) to test mice in a delayed alternation task (DAT) in a longitudinal manner. Mice were rewarded when they entered alternate sides of the maze on subsequent trials. Automation as well as connection of the F8M set-up with a home cage reduces experimenter interference and minimizes acute stress, thus making it suitable for longitudinal testing and facilitating clinical translation. In the present study, we monitored cognitive functioning of 2-month-old APPswe/PSEN1dE9 (APP/PS1) mice over a period of 4 months. The percentage of correct responses in the DAT did not differ between wild-type and transgenic mice from 2 to 6 months of age. However, 6-month-old mice displayed an increase in the number of consecutive incorrect responses. These results demonstrate the feasibility of longitudinal testing using an automated F8M and suggest that APP/PS1 mice are not impaired at delayed spatial alternation until 6 months of age under the current experimental conditions.

## Introduction

Alzheimer’s disease (AD) is one of the most prevalent neurodegenerative disorders and the most common cause of dementia (Blennow et al., 2006). Pathologically, the disease is characterized by extracellular amyloid beta plaques and intracellular tau tangles (Braak and Braak, 1990). These neuropathological hallmarks are especially pronounced in the hippocampal formation (Hyman et al., 1984; Braak and Braak, 1991). Accordingly, hippocampal atrophy has been detected at an early disease stage and correlates with changes in the cognitive status of patients, progressing from normal functioning to mild cognitive impairment (MCI) and AD (Ikeda et al., 1994; Jack et al., 2000; Mueller et al., 2010; Andrews et al., 2013). However, the relation between these neuropathological features and disease etiology is still unclear and, consequently, treatment that modifies early disease mechanisms is not yet available (Herrup, 2015; Masters et al., 2015; Makin, 2018). To obtain a better mechanistic understanding of events during the clinical (8–10 years), as well as the long preclinical and prodromal phases (up to 20 years before disease onset) in AD (Masters et al., 2015), animal research is essential. Moreover, it is crucial to perform animal studies in which disease-relevant cognitive functioning is monitored over extended periods of time.

In view of the longitudinal measurement of cognitive functioning in AD mouse models, behavioral paradigms commonly used in the AD field might pose several challenges. First, behavioral tasks are often not suitable for longitudinal testing. The stressful nature of a task, for instance the stress induced by the shock in contextual fear conditioning, can influence behavior in subsequent sessions, making it difficult to study disease progression in terms of cognitive decline. Alternatively, tasks may be labor-intensive and difficult to automate, thereby complicating longitudinal monitoring of task performance. Second, performance in some behavioral paradigms may be significantly influenced by non-cognitive factors. For example, in the Morris water maze, the animal learns to use distal visual cues in order to locate a submerged platform in an open swimming arena. However, because the water is unfamiliar and aversive to the mouse, anxiety plays an important role in addition to cognition (Wolfer et al., 1998). This complicates the interpretation of task performance in terms of cognitive functioning, especially as altered sensitivity to stress has repeatedly been reported for several AD mouse models (Dong et al., 2004; Jeong et al., 2006; Carroll et al., 2011; Rothman et al., 2012; Baglietto-Vargas et al., 2015; Stuart et al., 2017).

Addressing these issues, we considered an experimental protocol that allows for consecutive longitudinal testing of cognitive function, while minimizing acute stress that is imposed onto the animal. The proposed protocol is based on the delayed alternation task (DAT), an assay used to test working memory (Dudchenko, 2004). Working memory refers to the temporary storage and simultaneous processing of information (Baddeley, 1992). The DAT is commonly performed in a T-maze. Importantly, the DAT does not impose acute stress as it makes use of the natural tendency of rodents to alternate, which is thought to arise from their willingness to explore novel environments in search for information or resources, such as food, water, or shelter, that will aid their survival (Dember and Fowler, 1958; Lalonde, 2002). A delay can be built into the task by confining the animal in the base of the T for a certain amount of time, which increases task difficulty. The DAT can also be performed in a modified version of the T-maze, a figure-8-maze (F8M), in which the side arms are connected to the base of the maze, so that the animal can follow a unidirectional trajectory in the shape of an 8. The F8M minimizes experimenter intervention and increases throughput, thus potentially making the DAT suitable for automation and longitudinal testing. Complete automation using computer vision (Pedigo et al., 2006) even eliminates the presence of an experimenter. Connecting the F8M to the animals’ home cage (Schaefers and Winter, 2011) can further reduce animal handling. In accordance with the role of both the hippocampus and the prefrontal cortex in working memory, F8M studies have indicated involvement of these brain regions in the DAT (Pedigo et al., 2006; Ainge et al., 2007; Yoon et al., 2008; Pioli et al., 2014).

In the present study, we test the proposed F8M protocol using the APPswe/PSEN1dE9 (APP/PS1) mouse strain (line 85), a widely used mouse model of AD (Jankowsky et al., 2004; Radde et al., 2006). This is a double transgenic model that harbors the 695-amino acid mouse/human amyloid precursor protein (APP) transgene with the Swedish mutation as well as a mutant human presenilin 1 transgene (PSEN1/dE9). Both mutations are associated with early-onset AD. Amyloid beta plaques have been detected at 6 months of age (moa) in this mouse model (Jankowsky et al., 2004) and cognitive deficits have been reported in multiple behavioral tasks such as the Morris water maze (Cao et al., 2007), contextual fear conditioning (Cramer et al., 2012), novel object recognition (Guo et al., 2015; Petrov et al., 2015), and the hole-board maze (Reiserer et al., 2007; Hooijmans et al., 2009) at around this time. Furthermore, already at 3–4 months of age, dysfunction of hippocampal circuitry and associated memory decline have been detected in these mice (Park et al., 2006; Vegh et al., 2014; Hijazi et al., 2019). We therefore performed a longitudinal experiment in APP/PS1 mice at 2–6 moa to identify symptom progression as a function of age. When comparing task performance of APP/PS1 mice to wild-type control mice, we found similar response accuracy for both genotypes. However, the number of consecutive incorrect responses made by APP/PS1 mice was increased at 6 moa. In light of these findings, we discuss several advantages and limitations of the automated F8M test set-up.

## Materials and Methods

### Animals

Male APP/PS1 and APP/PS1-PV-Cre mice were used in this study. APP/PS1 mice [The Jackson Laboratory; strain B6C3-Tg(APPswe,PSEN1dE9)85Dbo/J with stock number 004462; MMRRC stock #34829] are double transgenic mice that express a chimeric human/mouse APP gene (Mo/HuAPP695swe) as well as a mutant human PS1 gene harboring a deletion of exon 9 (PS1dE9) under the control of a mouse prion protein promoter (MoPrP.Xho) (Jankowsky et al., 2001, 2003, 2004; Reiserer et al., 2007). APP/PS1-PV-Cre mice are a cross of APP/PS1 mice with PV-Cre mice [The Jackson Laboratory; Strain B6.129P2-Pvalbtm1(cre)Arbr/J with stock number 017320], which express Cre recombinase under the control of the endogenous parvalbumin (Pvalb) promoter. These mice were included to allow for future PV interneuron-specific interventions. In the absence of Cre-dependent interventions, APP/PS1-PV-Cre mice behave similar to APP/PS1 mice (Hijazi et al., 2019). Mouse lines were maintained on a C57BL/6JCrl background (Charles River Laboratories), and experiments were performed with individually-housed male mice. Wild-type and transgenic littermate mice were used in the study. Mice were kept on a reversed 12-h day–night cycle, with the dark phase starting at 9 am. Mice had *ad libitum* access to food, and during the training and test phases, they were water-deprived in the home cage for maximally 18 h preceding access to the F8M. All experiments were approved by the Central Committee for Animal Experiments (CCD) and the Animal Welfare Body of Vrije Universiteit Amsterdam in full compliance with the directive 2010/63/EU.

### Figure-8-Maze Apparatus and Data Collection

**FIGURE 1:**
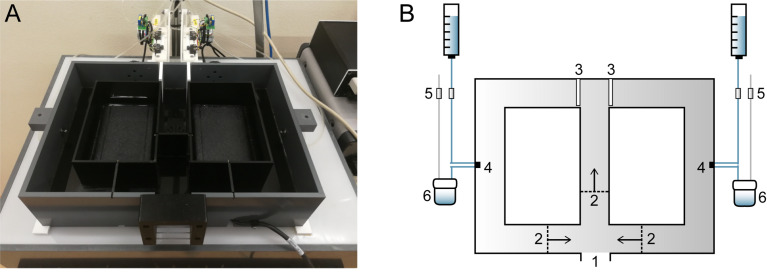
Home cage-based figure-8-maze (F8M) setup. **(A)** Photograph of the F8M. The maze is made of infrared-transmitting perspex (top lid not shown in photograph) and placed on top of an infrared light box. **(B)** Schematic drawing of the F8M. The F8M can be connected to the mouse’s home cage so that the mouse can enter the maze voluntarily (1). Once entered, three unidirectional swing doors (2) limit movement of the mouse to one direction as indicated by the arrows. The two motorized doors (3; shown in closed position) are under computer control. When the mouse makes a correct response, a sucrose water reward is delivered to either of two reward orifices (4) in precise preset quantities. Four computer-controlled valves (5) control the delivery of the reward, as well as its retraction by a vacuum pump into a container (6) when not consumed.

The F8M (24.3 cm × 33.3 cm × 8 cm) has 4 cm wide corridors and is made of black Perspex that transmits infrared light (Figure 1). An infrared light box is positioned beneath the maze, which allows for tracking of the animal with a camera that is located above the maze. The entrance of the maze can be connected to the animal’s home cage. Three swing doors surrounding the entrance open in one direction only, thus ensuring that the animal always moved through the maze in the same direction. Two motorized doors at the T-junction are controlled by the computer and open horizontally. Mice received a drop (18 μL) of water supplemented with 1% sucrose upon entering the correct maze arm. Two 12-V HDI valves (cat. no. LHDA1231415H, Denis de Ploeg, Netherlands), controlled by the computer, automate the water supply at each reward location. Opening the first valve allows the water droplet to be formed; opening the second valve retracts the previously formed drop by creating a vacuum. The maze was cleaned with ethanol in between sessions.

Using tracking software (Viewer17, Biobserve, Germany), the computer uses the camera input (cat. no. 18140P0005, Sunkwang Electronics, Korea) to control the maze’s motorized doors and valves based on the animal’s position within the maze. The software creates a time-stamped file with the executed commands and the animal’s responses and location.

### Behavioral Task

#### Experiment Phases

**FIGURE 2:**
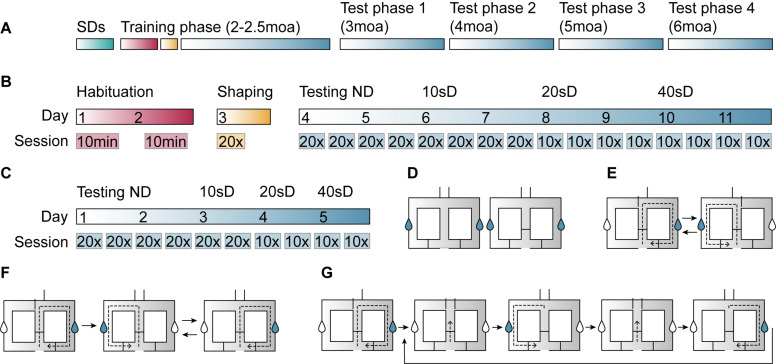
Illustration of the delayed alternation task (DAT) procedure in the figure-8-maze. **(A)** After being exposed to swing doors (SDs) in the home cage for 2 days (green), mice performed a DAT training phase at 2–2.5 moa and were tested at 3, 4, 5, and 6 moa. **(B)** In the training phase, mice were exposed to the maze during two habituation sessions (red), performed 20 shaping trials (yellow), and had four testing sessions at each of the four delays (no delay, 10-, 20-, and 40-s delay) (blue). **(C)** Test phases consisted of four no-delay sessions and two sessions of the 10-, 20-, and 40-s delays. **(D)** During habituation sessions, mice were free to explore the maze with the motorized doors open and either 2 (day 1, left image) or 3 (day 2, right image) SDs present. Sucrose-containing rewards (blue drops in graphical representation) could be obtained at either side of the maze. **(E)** During the shaping session, mice performed 20 trials of forced alternation. **(F)** No-delay sessions consisted of a forced run (left image), followed by 20 free run trials, whereby the animal was rewarded when entering the arm that had not been visited on the previous run. **(G)** In delay sessions, the animal was contained within the middle compartment (second and fourth image) for a period of 10, 20, or 40 s before the motorized doors would open and it could make a response.

The experiment consisted of a training phase and four test phases (Figure 2A). Two days prior to the training phase, two swing doors were placed in the animals’ home cage to familiarize mice with the doors. Then, mice underwent an 11-day training phase that consisted of habituation, shaping, and testing sessions. In the test phases following the training phase, the protocol was shortened to 5 days and consisted of testing sessions only.

#### Habituation

Mice were first habituated to the maze in two 10-min sessions on subsequent days (Figure 2B). During the first and second habituation session, two and three swing doors were present, respectively (Figure 2D). In both habituation sessions, all motorized doors were open. Sucrose-containing water rewards were provided at either side of the maze.

#### Shaping

After the habituation phase, mice underwent 20 shaping trials during which the right and left motorized doors were opened in alternating order (Figure 2B). Each trial consisted of the animal starting in the center zone, entering the left or right arm of the maze, and then returning to the center zone (Figure 2E).

#### Testing

First, mice were tested in the F8M without a delay (Figure 2B). The session started with a rewarded forced-choice run into the right arm of the maze (Figure 2F). All 20 subsequent trials were free-choice runs in which the correct response was for the animal to choose the opposite arm from the one it had visited on the previous trial. A correct response was rewarded with a sucrose-containing water reward, whereas no reward was administered upon an incorrect response. During these no-delay sessions, the motorized doors would open when the animal entered the center zone and they would close once the animal had moved into either of the two side arms. The animal performed four sessions of one forced run followed by 20 free-run trials over 2 days. Performance was calculated as the percentage of correct responses per 20 free-choice trials.

Next, mice were tested with three different delay intervals built into the task: a 10-, 20-, and 40-s delay (Figure 2B). The delay, during which the two motorized doors remained closed, started when the animal entered the center zone (Figure 2G). When the delay time ended, both motorized doors opened and the animal could make its choice. Similar to the no-delay sessions, delay sessions started with a forced-choice run into the right arm of the maze, followed by 20 open-choice trials. During the training phase, mice would perform four sessions per delay interval divided over 2 days. These sessions consisted of 20 open-choice trials for the 10-s delay sessions, and 10 open-choice trials for the 20- and 40-s delay sessions. During the test phases, mice carried out only two sessions per delay (Figure 2C). Every test phase started with no-delay testing sessions to ensure that later task performance, when delays were introduced, would reflect the animals’ ability to alternate rather than the ability to remember task rules.

### Data Analysis

Data were analyzed using MATLAB R2017b (MathWorks) and visualized using Prism 8.2.1 (GraphPad Software). Statistical testing was performed in Prism using a two-factor repeated measures ANOVA or mixed-effects analysis, combined with a Geisser-Greenhouse correction when the data were non-spherical. When significant differences (*p* < 0.05) were found, *post hoc* comparisons were performed using Bonferroni’s multiple comparisons test. For the analysis of response latencies, outlier values were removed using the ROUT method with Q set at 0.1% (Motulsky and Brown, 2006). Statistical details of experiments can be found in the respective results sections and in tables. Results and graphs report mean ± SEM. The number of animals used in each experiment is provided in the figure legends. Sessions in which mice did not complete 20 trials (for no-delay and 10-s delay) or 10 trials (for 20 and 40-s delay) within 1 h were excluded from analysis.

## Results

### Response Accuracy

**FIGURE 3:**
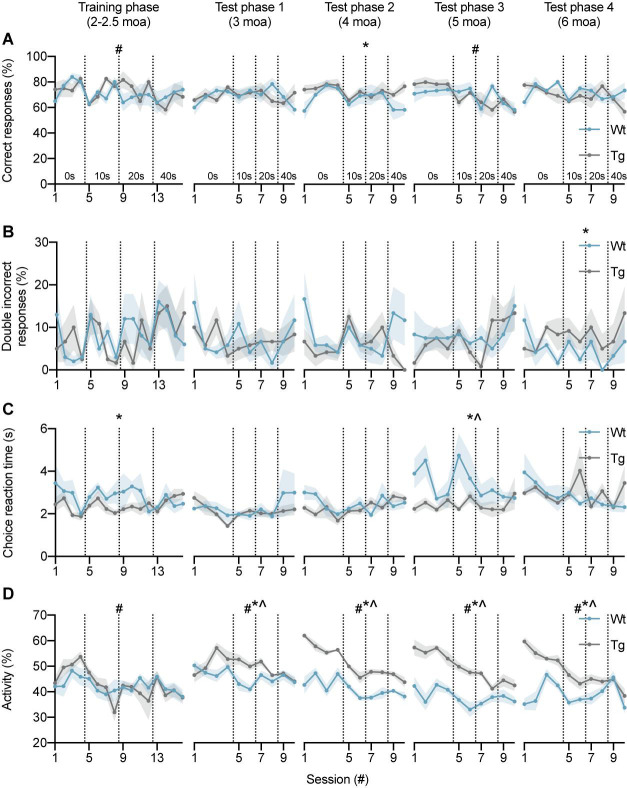
DAT performance of wild-type and APP/PS1 mice at 2–2.5, 3, 4, 5, and 6 moa. **(A)** Response accuracy of wild-type (*n* = 6, blue) and APP/PS1 (*n* = 6, gray) mice during training and test phases. During the training phase, two-way ANOVA showed an effect of session (#), with significant differences between sessions 3–5, 4–5, and 5–8. In test phase 2, a main effect of genotype (*) was observed. Analysis of test phase 3 showed a main effect of session, with significant differences between sessions 4–9 and 6–10. **(B)** APP/PS1 mice showed an increase in the percentage of consecutive incorrect responses in test phase 4 at 6 moa. **(C)** Choice reaction time of APP/PS1 mice was significantly lower compared to wild-type controls in the training phase and test phase 3. **(D)** At 3, 4, 5, and 6 moa, APP/PS1 mice were more active than wild-type mice. Vertical lines indicate the start of a new delay interval. #main effect of session, *p* < 0.05; *main effect of genotype *p* < 0.05; ^interaction effect *p* < 0.05.

We first determined whether APP/PS1 and wild-type mice were both able to learn the DAT using the automated F8M protocol. During the training phase at 2–2.5 moa, correct responses reached 82.5 ± 2.5% and 80.0 ± 5.5% for APP/PS1 and wild-type mice, respectively, during the fourth no-delay testing session (Figure 3A), indicating that animals from both genotypes had successfully learned the task. Next, to determine whether performance was dependent on session or genotype during the training phase, a two-way repeated measures ANOVA was performed (Table 1). A significant main effect of session [*F*_(5.39, 48.48)_ = 2.43, *p* = 0.04] was found, with Bonferroni’s multiple comparisons test showing significant differences in response accuracy between session 3–5 (*p* = 0.032), 4–5 (*p* = 0.0021), and 5–8 (*p* < 0.001), indicating an overall decrease in task performance upon introduction of the 10-s delay (session 4–5) and an improvement in performance toward the end of the 10-s delay sessions (session 5–8). Whereas test phase 1 at 3 moa did not reveal a main or interaction effect, test 2 at 4 moa showed a main effect of genotype [*F*_(1,10)_ = 6.21, *p* = 0.03], indicating improved performance of APP/PS1 mice compared to wild-type controls. Test phase 3 at 5 moa revealed a main effect of session [*F*_(4.57,44.71)_ = 4.4, *p* = 0.003]. Bonferroni’s multiple comparisons test showed differences in response accuracy between session 4–9 (*p* = 0.047) and 6–10 (*p* = 0.012), indicating a decrease in performance during the 40-s delay interval (session 9 and 10) compared to 0- and 10-s delay intervals. Lastly, test phase 4 at 6 moa did not reveal any significant differences. These results indicate that mice learnt the DAT in the current F8M set-up with a trend for a decrease in response accuracy as delay intervals were increased. Except for test phase 2, during which APP/PS1 mice performed better than wild-type mice, both genotypes performed the task at similar levels of response accuracy. To exclude the possibility that levels of response accuracy were influenced by the fact that these animals had been repeatedly tested over the course of several months, an additional group of mice was tested at 6 moa only (Supplementary Figure 1). No significant differences in response accuracy were found (Supplementary Table 1). Even though the group size was limited, these data suggest that also in that absence of repeated testing over the course of several months, 6-month-old mice of both genotypes can perform the task.

**TABLE 1:** Response accuracy.

	Session	Genotype	Session × Genotype
Training	*F*_(5.39,48.48)_ = 2.43, *p* = 0.04	*F*_(1,9)_ = 0.11, *p* = 0.75	*F*_(15,135)_ = 1.41, *p* = 0.15
Test 1	*F*_(5.27,52.71)_ = 1.24, *p* = 0.30	*F*_(1,10)_ = 0.001, *p* = 0.98	*F*_(9,90)_ = 1.33, *p* = 0.23
Test 2	*F*_(4.71,47.07)_ = 2.19, *p* = 0.075	*F*_(1,10)_ = 6.21, *p* = 0.03	*F*_(9,90)_ = 1.14, *p* = 0.35
Test 3	*F*_(4.57,44.71)_ = 4.4, *p* = 0.003	*F*_(1,10)_ = 0.002, *p* = 0.96	*F*_(9,88)_ = 1.68, *p* = 0.11
Test 4	*F*_(4.46,44.61)_ = 1.43, *p* = 0.24	*F*_(1,10)_ = 2.71, *p* = 0.13	*F*_(9,90)_ = 1.83, *p* = 0.074

### Error Perseveration

Next, the percentage of consecutive incorrect responses was analyzed since this parameter has been shown to be increased by hippocampal inactivation (Yoon et al., 2008) (Figure 3B). No main or interaction effects were found in the training phase, test phase 1, test phase 2, or test phase 3 (Table 2). Test phase 4, however, did show a main effect of genotype [*F*_(1,10)_ = 5.00, *p* = 0.049], indicating that APP/PS1 mice make more consecutive incorrect responses than wild-type mice at 6 moa. Test phase 4 did not reveal a main effect of session or an interaction effect. These findings suggest that the percentage of consecutive incorrect responses might be a sensitive measure of hippocampal impairment in APP/PS1 mice.

**TABLE 2:** Error perseveration.

	Session	Genotype	Session × Genotype
Training	*F*_(4.35,39.20)_ = 1.92, *p* = 0.12	*F*_(1,9)_ = 0.019, *p* = 0.89	*F*_(15,135)_ = 1.01, *p* = 0.45
Test 1	*F*_(3.64,36.35)_ = 1.17, *p* = 0.34	*F*_(1,10)_ = 0.03, *p* = 0.86	*F*_(9,90)_ = 0.75, *p* = 0.66
Test 2	*F*_(4.59,45.89)_ = 1.36, *p* = 0.26	*F*_(1,10)_ = 2.40, *p* = 0.15	*F*_(9,90)_ = 1.80, *p* = 0.080
Test 3	*F*_(4.54,49.46)_ = 1.59, *p* = 0.18	*F*_(1,98)_ = 0.50, *p* = 0.48	*F*_(9,98)_ = 0.78, *p* = 0.63
Test 4	*F*_(4.19,41.91)_ = 1.12, *p* = 0.36	*F*_(1,10)_ = 5.00, *p* = 0.049	*F*_(9,90)_ = 0.73, *p* = 0.68

### Choice Reaction Time

Choice reaction time (CRT) was defined as the time period between the opening of the motorized doors and the moment the animal enters either maze arm. CRT showed a main effect of genotype during the training phase [*F*_(1,9)_ = 6.76, *p* = 0.029], indicating a decrease in CRTs for APP/PS1 mice (Figure 3C). No significant differences were found in test phase 1 and test phase 2 (Table 3). Test phase 3 revealed a main effect of genotype [*F*_(1,10)_ = 5.50, *p* = 0.041] and an interaction effect [*F*_(9,84)_ = 2.05, *p* = 0.043]. Test phase 4 did not show any significant differences. Overall, these results indicate that APP/PS1 mice had reduced CRTs during the training phase and test phase 3.

**TABLE 3:** Choice reaction time.

	Session	Genotype	Session × Genotype
Training	*F*_(4.54,39.97)_ = 1.30, *p* = 0.28	*F*_(1,9)_ = 6.76, *p* = 0.029	*F*_(15,132)_ = 1.12, *p* = 0.34
Test 1	*F*_(1.50,14.32)_ = 1.49, *p* = 0.25	*F*_(1,10)_ = 0.44, *p* = 0.52	*F*_(9,86)_ = 0.76, *p* = 0.65
Test 2	*F*_(3.42,33.06)_ = 1.86, *p* = 0.15	*F*_(1,10)_ = 0.81, *p* = 0.39	*F*_(9,87)_ = 1.79, *p* = 0.082
Test 3	*F*_(2.90,27.11)_ = 2.010, *p* = 0.14	*F*_(1,10)_ = 5.50, *p* = 0.041	*F*_(9,84)_ = 2.05, *p* = 0.043
Test 4	*F*_(3.11,30.74)_ = 1.50, *p* = 0.23	*F*_(1,10)_ = 0.15, *p* = 0.71	*F*_(9,89)_ = 1.56, *p* = 0.14

### Activity

To determine whether differences in CRT between wild-type and APP/PS1 mice were related to changes in general activity levels, we analyzed overall activity of the mice (Figure 3D). During the training phase, a main effect of session [*F*_(4.1,41)_ = 4.0, *p* = 0.0073] was found (Table 4). Main effects of session and genotype, as well as interaction effects, were found in test phase 1 [Session *F*_(4.2,42)_ = 3.4, *p* = 0.0148; Genotype *F*_(1,10)_ = 11, *p* = 0.0087; Session × Genotype interaction *F*_(9,90)_ = 3.0, *p* = 0.0034], test phase 2 [Session *F*_(3.8,38)_ = 16, *p* < 0.0001; Genotype *F*_(1,10)_ = 115, *p* < 0.0001; Session × Genotype interaction *F*_(9,90)_ = 3.2, *p* = 0.0021], test phase 3 [Session *F*_(4.0,40)_ = 8.2, *p* < 0.0001; Genotype *F*_(1,10)_ = 50, *p* < 0.0001; Session × Genotype interaction *F*_(9,90)_ = 3.0, *p* = 0.0035], and test phase 4 [Session *F*_(3.1,31)_ = 12, *p* < 0.0001; Genotype *F*_(1,10)_ = 26, *p* = 0.0004; Session × Genotype interaction *F*_(9,90)_ = 9.2, *p* < 0.0001]. These findings show that APP/PS1 mice are significantly more active than wild-type mice in all test phases.

**TABLE 4:** Activity.

	Session	Genotype	Session × Genotype
Training	*F*_(4.1,41)_ = 4.0, *p* = 0.0073	*F*_(1,10)_ = 0,049, *p* = 0.83	*F*_(15,148)_ = 1.2, *p* = 0.29
Test 1	*F*_(4.2,42)_ = 3.4, *p* = 0.0148	*F*_(1,10)_ = 11, *p* = 0.0087	*F*_(9,90)_ = 3.0, *p* = 0.0034
Test 2	*F*_(3.8,38)_ = 16, *p* < 0.0001	*F*_(1,10)_ = 115, *p* < 0.0001	*F*_(9,90)_ = 3.2, *p* = 0.0021
Test 3	*F*_(4.0,40)_ = 8.2, *p* < 0.0001	*F*_(1,10)_ = 50, *p* < 0.0001	*F*_(9,90)_ = 3.0, *p* = 0.0035
Test 4	*F*_(3.1,31)_ = 12, *p* < 0.0001	*F*_(1,10)_ = 26, *p* = 0.0004	*F*_(9,90)_ = 9.2, *p* < 0.0001

## Discussion

We designed an automated F8M to monitor cognitive function in AD mice in a longitudinal manner. Using this set-up, we tested APP/PS1 mice from 2 to 6 moa to determine the feasibility of longitudinal testing and the sensitivity of the task to monitor symptom progression as a function of age. Starting at 2 moa, mice were trained to perform a DAT with a 0-, 10-, 20-, or 40-s delay, after which they were tested once every 4 weeks until they were 6 moa. All mice learnt the task. While we did not find an age-dependent decrease in choice accuracy, APP/PS1 mice made more consecutive incorrect responses than wild-type mice at 6 moa. The current study demonstrates the feasibility of longitudinal monitoring of cognitive function using a DAT protocol in an automated F8M. Even though longitudinal studies on cognitive function in AD mouse models, and spatial memory in specific, have been performed previously, for example, assessing water maze performance of APP/PS1 mice (Ferguson et al., 2013), they are sparse.

Both wild-type and APP/PS1 mice learnt the task equally well. Response accuracies of APP/PS1 mice and wild-type controls (82.5 and 80%, respectively) during the last no-delay test session of the training phase were comparable to the percentages of correct responses reported in other F8M studies using mice (Schaefers and Winter, 2011; Shoji et al., 2012). The response accuracy tended to decrease with the introduction of delays into the task, indicating an increase in memory load as the mice had to keep previous arm entries online for an extended period of time. This is in line with previous studies showing a decrease in the percentage of correct responses with increasing delays (Pedigo et al., 2006; Schaefers and Winter, 2011; Shoji et al., 2012). Whereas we did not find impaired DAT response accuracy by APP/PS1 mice, we did observe that they made more consecutive incorrect responses at 6 moa. The number of consecutive incorrect responses has previously been linked to hippocampal functioning (Yoon et al., 2008). When the dorsal hippocampus (dHPC) was inactivated using muscimol, rats showed an increase in the percentage of double incorrect responses across delays compared to when the medial prefrontal cortex (mPFC) was inactivated. Thus, these findings suggest that the increase in the number of consecutive incorrect responses observed here might be an early measure of hippocampal dysfunction in the APP/PS1 mouse model. However, it is unclear whether perseveration of choice response reflects a memory deficit or whether it results from a change in behavior, for example, a change in the mice’s natural tendency to alternate. It would be interesting to see whether the increase in the number of double incorrect responses persists and increases with age. Besides an increase in the number of consecutive incorrect responses, we also found that CRT was decreased in APP/PS1 mice compared to wild-type controls during the training phase and test phase 3. One might hypothesize that shorter response latencies simplify the DAT for APP/PS1 mice by reducing working memory load, thereby masking subtle memory deficits at early disease stages. Even though we cannot exclude this possibility, other experiments suggest that decreased latencies do not necessarily translate to better performance. CaMKII^+/–^ mice, for instance, show a decreased correct response rate compared to wild-type controls, even though their response latencies are decreased (Shoji et al., 2012). Potentially explaining the reductions in CRTs, we also found increased levels of activity for APP/PS1 mice. An increase in general activity levels of APP/PS1 mice has been reported previously (Lalonde et al., 2005; Filali et al., 2011; reviewed by Lalonde et al., 2012). It is not yet clear what causes hyperactivity in APP/PS1 mice. Hyperactivity might be related to hippocampal changes as mice with hippocampal lesions (Kleinknecht et al., 2012) or NMDA receptor blockade (Stiedl et al., 2000) exhibit increased locomotor activity with memory impairments. In addition, hyperactivity might be linked to a reduction in GABAergic neurotransmission, since hyperactivity emerges at the same time as seizure activity in APP_751_SWE mice (Dumont et al., 2004) and GABA_*A*_ receptor antagonists injected into the hippocampus increase motor activity in rats (Bast et al., 2001). Changes in activity in mice may be reminiscent of neuropsychiatric symptoms in patients with dementia, such as apathy and agitation (Lyketsos et al., 2000, 2002). Mice have been suggested to be hypoactive as a result of apathy or hyperactive due to agitation (Lalonde et al., 2012). The hyperactivity observed in APP/PS1 mice could be related to an attention deficit. The literature on attention in AD mouse models is inconclusive (Romberg et al., 2013a; Shepherd et al., 2016), with some studies showing reduced attention in AD mice (Romberg et al., 2011, 2013b) and others showing no deficit (Bharmal et al., 2015; Kent et al., 2018; Shepherd et al., 2021). Even though Shepherd et al. (2021) did not observe reduced accuracy in the five-choice serial-reaction time task in 9–11-month-old APP/PS1 mice, they did not also find a change in general activity. It would be interesting to further investigate the relationship between hyperactivity and attention in future experiments.

Comparison of F8M performance of APP/PS1 mice to their wild-type littermates highlights several advantages as well as limitations of the current task set-up and testing protocol. APP/PS1 mice did not show impaired response accuracy at any delay at any age. We had hypothesized an age-dependent decline in response accuracy, considering previously reported hippocampal spatial memory deficits in APP/PS1 mice in the Morris water maze, radial arm water maze, and contextual fear conditioning at an early disease stage (Park et al., 2006; Vegh et al., 2014; Hijazi et al., 2019) and the role of the hippocampus in delayed alternation, specifically at non-zero delays (Wan et al., 1994; Hampson et al., 1999; Steele and Morris, 1999; Zhang et al., 2013). In specific, the F8M-based DAT has been shown to be hippocampus-dependent (Yoon et al., 2008; Pioli et al., 2014) and hippocampal lesions affect task performance in a delay-dependent manner (Ainge et al., 2007). Hippocampus lesioned rats showed a deficit when a 2- or 10-s delay was introduced into the task, but not in the absence of a delay. Our results here suggest that in contrast to the reported impairments in long-term spatial and contextual memory, short-term working memory as measured by DAT response accuracy in the F8M is not yet affected in APP/PS1 mice up to 6 moa. Other studies investigating spatial working memory in APP/PS1 mice have shown variable results, with some studies reporting working memory deficits (Kim et al., 2015; Wang et al., 2017) and others not finding a difference between wild-type and transgenic animals (Lalonde et al., 2004; Reiserer et al., 2007; Harrison et al., 2009). Whereas spontaneous alternation is a commonly used measure of working memory, studies on rewarded alternation with variable delay intervals in the APP/PS1 mouse model are sparse.

There are several potential explanations for the absence of an age-related decline in F8M response accuracy. First, it might be that even though the delayed alteration in the F8M is hippocampus-dependent, the hippocampal dysfunction previously observed in APP/PS1 mice is not sufficient to impair task performance. As working memory is thought to require communication between the hippocampus and prefrontal cortex (Jin and Maren, 2015), cortical mechanisms might be able to compensate for (mild) hippocampal dysfunction during a short-term working memory task.

Second, in the current test set-up, mice may be able to solve the F8M using strategies that are not hippocampus-dependent. One possibility is that, as the maze is opaque and testing occurs during the dark phase, mice might use egocentric navigation strategies that are not dependent on the hippocampus. Egocentric navigation makes use of internal cues (e.g., limb movement for speed, direction, and turns), optic flow, and signposts (Vorhees and Williams, 2014), as opposed to allocentric navigation, where space is encoded on the basis of distal cues (landmarks) and the relationship between those cues. Egocentric navigation seems to preferentially involve the dorsal striatum and connected structures, whereas allocentric navigation depends on the entorhinal cortex-hippocampal system (for review, see Buzsaki and Moser, 2013). However, these two systems are also thought to interact (Goodroe et al., 2018). For instance, hippocampal episodic memory mechanisms could play a role in egocentric route-oriented memory, as retrieval of routes can be considered as the retrieval of separate spatiotemporal events. Several other F8M studies have also tested animals during the dark phase (Pedigo et al., 2006; Yoon et al., 2008; Schaefers and Winter, 2011). Interestingly, Mair et al. (1998) tested hippocampus-lesioned rats on a delayed non-match-to-sample task in a three-arm radial maze with lights on and lights off. Lesioned rats showed a delay-dependent deficit that was present both when lights were on and off, suggesting that also in the dark the hippocampus is important for task performance. For future studies, it will be of interest to use a transparent or open version of the F8M so that AD mice can be tested both with lights on and off in order to distinguish between ego- and allocentric strategies, in particular because both types of navigation strategies have been reported to be affected in people with MCI or AD (Serino et al., 2015; Boccia et al., 2016; Tu et al., 2017; Coughlan et al., 2018). Another possibility is that mice may have performed the DAT using hippocampus-independent stimulus-response (S-R) associations rather than spatial learning. In S-R learning, also known as habit learning, mice respond to a stimulus (e.g., the T-junction of the maze) with a certain response (e.g., turn right) (Knowlton and Patterson, 2018). Several factors in the current task set-up may have promoted habit formation. First, the task consisted of continuous alternation (rather than having a forced run followed by a free run). Second, every testing period started with four no-delay sessions, and third, mice were repeatedly tested over several months. Habit formation depends primarily on the striatum (Packard et al., 1989; Packard and McGaugh, 1992; McDonald and White, 1994; Moussa et al., 2011; Smith and Graybiel, 2013), a brain structure that is only affected at a later stage of AD (Thal et al., 2002). However, when we trained an additional group of mice at 6 moa only, APP/PS1 mice still performed at wild-type levels, suggesting that mice of both genotypes can perform the task in the absence of procedural memory being formed due to repeated testing over the course of several months. In addition, we would not expect habit formation to be sufficient to perform the DAT with extended delays, as these delays interrupt the execution of continuous habitual motor programs. Nevertheless, a forced run-free run protocol as well as fewer or no no-delay sessions might be able to minimize the formation of procedural memory in future experiments.

Finally, a potential explanation for the absence of a memory deficit in the F8M is that the current task set-up minimizes stress. Several aspects of the task minimize the acute stress that is imposed on the animal. First of all, all testing is performed without experimenter intervention. Second, habituation and shaping sessions habituate the mice to the maze apparatus so that the maze environment is no longer novel and stressful. Third, the animals enter the maze voluntarily during their dark phase, when C57BL/6J mice are naturally most active and intrinsically motivated to explore as nocturnal species (Hager et al., 2014; Loos et al., 2014). Fourth, the task itself does not impose acute stress. Several studies reporting deficits in APP/PS1 mice, especially at young ages, have used tests that involve acutely inflicted stress, such as the Morris water maze and contextual fear conditioning. In these tasks, it has been shown that non-cognitive factors, such as anxiety, can influence task performance (Wolfer et al., 1998; Gerlai et al., 2002). Since multiple studies have suggested altered sensitivity to stress in AD mouse models (Dong et al., 2004; Jeong et al., 2006; Carroll et al., 2011; Rothman et al., 2012; Baglietto-Vargas et al., 2015; Stuart et al., 2017), deficits that have been reported at early disease stages may reflect an interaction between altered stress levels and spatial memory. Even though in the current study the task set-up itself minimizes stress, we cannot exclude the possibility that the periods of water deprivation used to motivate the mice to perform the DAT may have been stressful for the mice. To minimize stress due to water deprivation, mice had access to water during their dark phase, which is the period in which they naturally drink most (Kiryk et al., 2020). We did not detect changes in body weight due to water deprivation (Supplementary Figure 2), and mice were checked on a daily basis with no signs of stress or compromised health being observed. For future studies, it would be ideal to permanently connect the home cages to an F8M apparatus such that water deprivation is no longer necessary.

A limitation of the current study is that the F8M protocol has not been directly compared to other test procedures that could be used longitudinally. An increasingly used method to test learning and memory in mice is the automated touchscreen platform (Horner et al., 2013). Similar to the F8M procedure described here, touchscreen tasks minimize stress, allow for a high degree of automation and standardization, and thus facilitate longitudinal testing. In addition, they have been successfully used to detect early cognitive deficits starting at 3 moa in APP/PS1-21 mice (Van den Broeck et al., 2021), suggesting higher sensitivity than the DAT. The preferred use of different procedures will depend on the aim of the study. An advantage of the current task set-up is that the animal is confined to a spatial compartment during the delay phase of the DAT, thus making it difficult for the mouse to encode the correct choice option by the position of its body. In addition, when performing *in vivo* measurements during task performance, i.e., local field potential (LFP) recordings, neural activity can be linked to specific cognitive processes (i.e., keeping online a previous arm entry and decision making) taking place at particular locations in the maze to better dissect processing steps during precise moments of the task.

To conclude, we designed a DAT protocol for longitudinal testing in an automated F8M, which we tested by comparing task performance between APP/PS1 and wild-type mice over a 4-month period. We found similar response accuracy for wild-type and APP/PS1 mice, but an increase in the number of consecutive incorrect responses for APP/PS1 mice at 6 moa. How relevant these findings are for AD remains a matter of speculation. AD patients have been shown to be impaired at a DAT based on the animal DAT (Freedman and Oscar-Berman, 1986; Bhutani et al., 1992; Collette et al., 1999). In addition, during the clinical phase as well as in MCI, patients have been reported to have compromised working memory (Kirova et al., 2015; Garcia-Alvarez et al., 2019). The absence of a genotype difference in the percentage of correct responses might suggest that our mice are still too young to detect these (pre)clinical symptoms. Irrespective of how exactly DAT phenotypes translate to human AD, the added value of the current set-up and protocol is that it allows for longitudinal testing and keeps acute stress imposed onto the animals low, due to the high degree of maze automation and the connection of the maze to the home cage. Longitudinal testing is especially important in AD as the disease is characterized by progressive loss of cognition and has a long preclinical and prodromal phase. Longitudinal studies allow for a better understanding of disease mechanisms in relation to symptom onset and progression. In addition, they provide advantages to translational AD research. Longitudinal screening can facilitate linking the different stages of cognitive dysfunction in animal models to the various phases of cognitive decline observed in AD patients. Moreover, it could aid in identifying risk and/or protective factors in the progression of MCI to AD and in predicting the effectiveness of potential treatments in mitigating or preventing cognitive decline.

## Data Availability Statement

The raw data supporting the conclusions of this article will be made available by the authors, without undue reservation.

## Ethics Statement

The animal study was reviewed and approved by the Central Committee for Animal Experiments (CCD) and Animal Welfare Body of Vrije Universiteit Amsterdam.

## Author Contributions

FH, SP, OS, AS, and RK designed the project and wrote the manuscript. FH and SP performed the experiments and did the analysis. All authors contributed to the article and approved the submitted version.

## Conflict of Interest

The authors declare that the research was conducted in the absence of any commercial or financial relationships that could be construed as a potential conflict of interest.
