# Supplementary material for: Longitudinal Assessment of Working Memory Performance in the APPswe/PSEN1dE9 Mouse Model of Alzheimer’s Disease Using an Automated Figure-8-Maze
Source: Front Behav Neurosci. 2021 May 13;15:655449. doi: 10.3389/fnbeh.2021.655449 (PMC8155296; doi:10.3389/fnbeh.2021.655449)
Supplement: Supplementary file 1 [file Image_1.pdf]

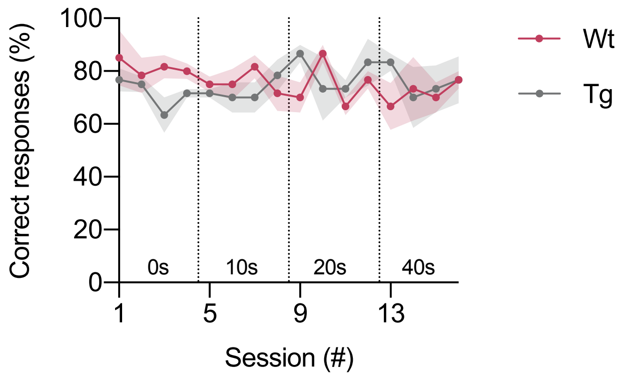

Supplementary Figure 1. Response accuracy does not differ between APP/PS1 and wild-type animals when tested at 6 months of age (moa) only. No significant differences were detected between APP/PS1 (n=3, grey) and wild-type (n=3, red) mice in choice accuracy irrespective of delay interval (0 – 40 s).

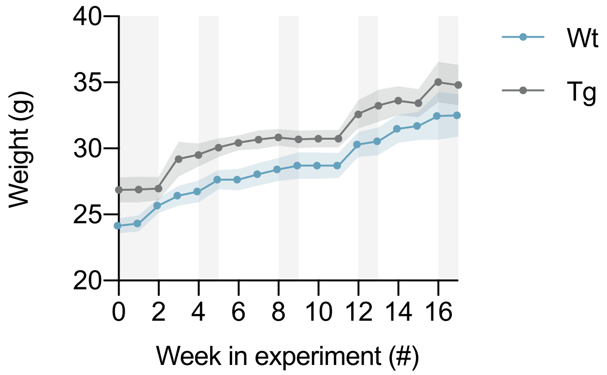

Supplementary Figure 2. Body weight of wild-type (n=6) and transgenic (n=6) mice during the experiment, as measured weekly from 2 until 6 moa. Areas shaded in grey are the training and test phases, and thus contain periods of water deprivation. Weighing at week 0 was performed prior to the start of the experiment. Two-factor ANOVA showed a significant main effect of time ($F_{(2.7,27)}=50$, $p<0.0001$), but no main effect of genotype nor an interaction effect.
