# Supplementary material for: Longitudinal Assessment of Working Memory Performance in the APPswe/PSEN1dE9 Mouse Model of Alzheimer’s Disease Using an Automated Figure-8-Maze
Source: Front Behav Neurosci. 2021 May 13;15:655449. doi: 10.3389/fnbeh.2021.655449 (PMC8155296; doi:10.3389/fnbeh.2021.655449)
Supplement: Supplementary file 2 [file Table_1.pdf]

Supplementary Table 1. Response accuracy of 6-month-old mice.

	**Session**	**Genotype**	**Session × Genotype**
**Training**	$F_{(2.6,10)}$=0.52, p=0.66	$F_{(1,4)}$=0.25, p=0.65	$F_{(15,60)}$=1.2, p=0.29
